# Supplementary material for: Electron tunneling at the molecularly thin 2D perovskite and graphene van der Waals interface
Source: Nat Commun. 2020 Oct 30;11:5483. doi: 10.1038/s41467-020-19331-6 (PMC7599242; doi:10.1038/s41467-020-19331-6)
Supplement: Supplementary file 1 — Supplementary Information [file 41467_2020_19331_MOESM1_ESM.pdf]

Supplementary Information

Electron Tunneling at the Molecularly Thin 2D Perovskite and Graphene Van der Waals Interface

Leng *et al*

Contents:

Supplementary Figures 1-13

Supplementary Tables 1-2

Supplementary References 1-16

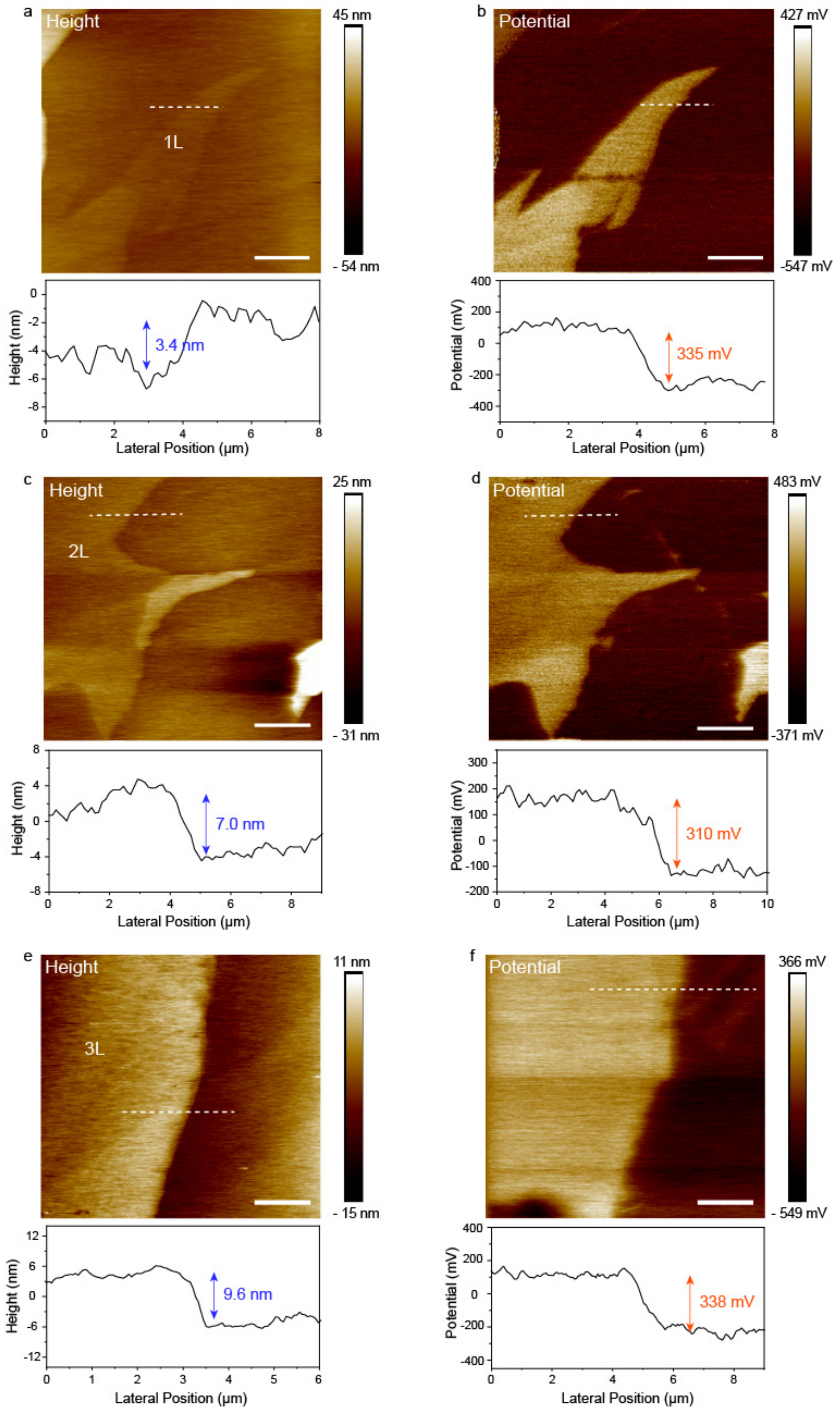

Supplementary Fig. 1 Thickness dependent KPFM measurements of n = 4 RPP flakes on G substrate. a, **c**, **e**, AFM images of exfoliated monolayer (**a**), bilayer (**c**) and trilayer (**e**) n = 4 RPP flakes on G substrate. **b**, **d**, **f**, Corresponding KPFM measurements of monolayer (**b**), bilayer (**d**) and trilayer (**f**) n = 4 RPP flakes on G. The external voltage is applied to the sample with the tip grounded. Scale bars (**a-d**), 6 μm; (**e**,**f**), 3 μm.

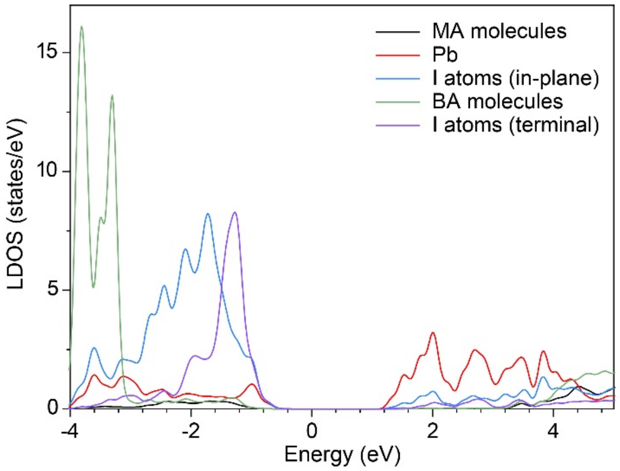

Supplementary Fig. 2 LDOS analysis of the top layer of n = 4 RPP. Based on LDOS result, the MA and BA organic molecules are almost invisible at the empty state STM image (tunnelling into conduction band between +1 to +3 eV) under small bias voltage. On the other hand, the filled state STM image (tunnelling from valence band) is a convolution of electron tunnelling from Pb, I and organic molecules.

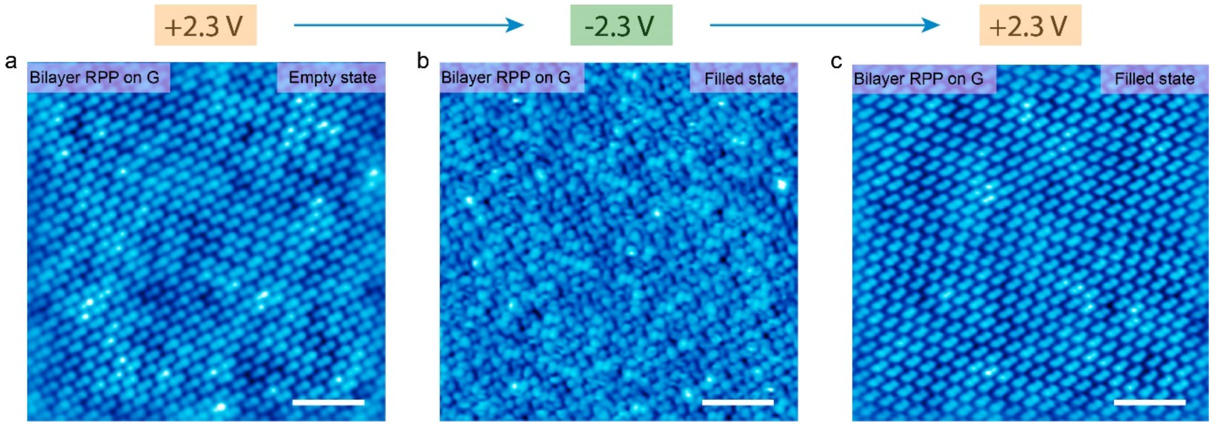

Supplementary Fig. 3 STM images of bilayer RPP (n = 4) at different bias sequence. a, STM image scanning under positive bias (empty state). **b**, The same scanning area in (**a**) under negative bias (filled state). **c**, The same scanning area in (**a**) under positive bias (empty state). The filled state image looks less ordered due to coupling of tunnelling electrons with vibronic motions of the organic cations. This is only dynamic disorder as order is recovered upon switching to positive bias. Scale bars, 4 nm (**a**-**c**).

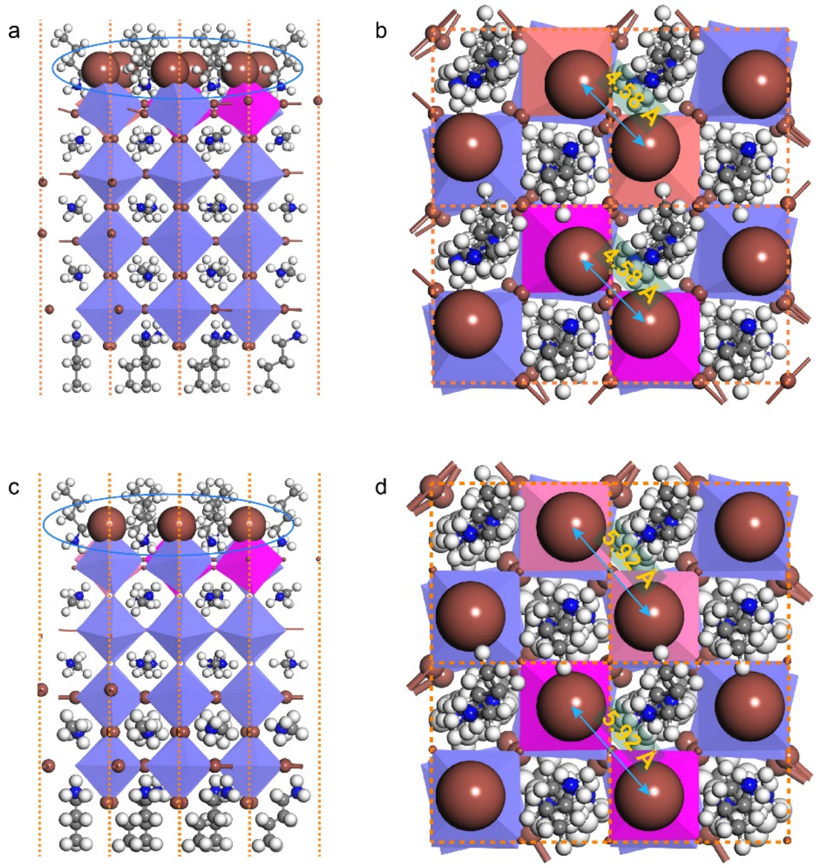

Supplementary Fig. 4 The calculated "paired" and "non-paired" structures in monolayer n = 4 RPP. a, The side view of paired structure with the nearest neighbour iodine atoms (brown ball) forming a paired structure. **b**, The top view of paired structure with distance of 4.58 Å of two nearest paired terminal iodine atoms; pairing occurs upon relaxation; **c**, The side view of unpaired structure based on non-relaxed surface. **d**, The top view of non-paired structure with distance of 5.92 Å of two iodine atoms. Brown ball, I; white ball, H; Blue ball, N; Grey ball, C; Octahedron, PbI_6.

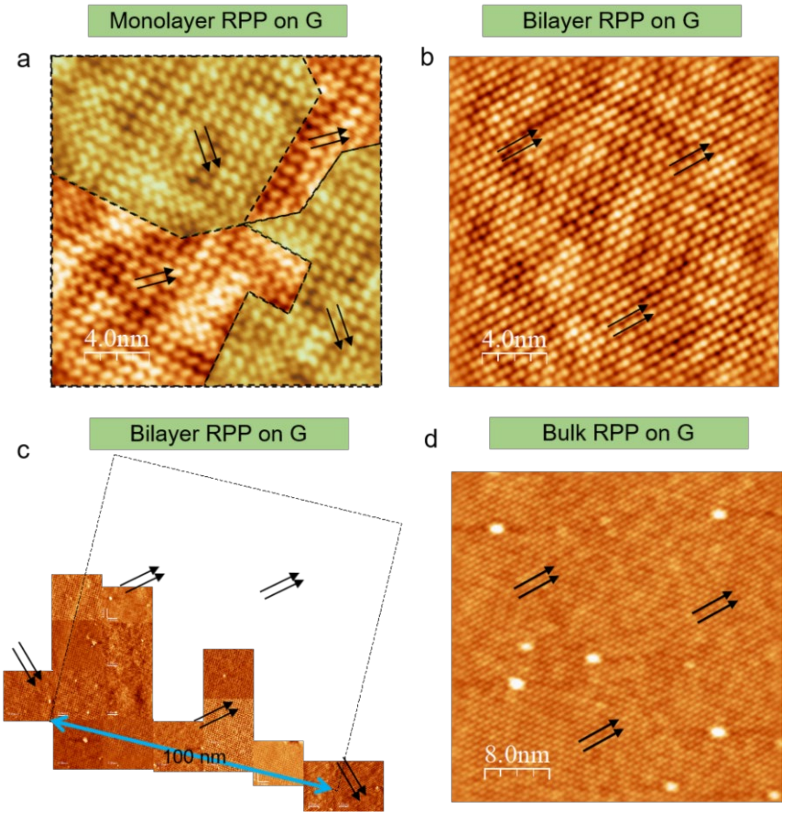

Supplementary Fig. 5 The single domain size in different thickness of RPP on graphene substrate. **a**, STM image of monolayer RPP on G with the domain size outlined by the dash line. **b**, Single domain of bilayer RPP on G. **c**, The width of 100 nm domain size in bilayer RPP. **d**, Single domain of bulk RPP on G. The orientation of the "paired" alignment is indicated as black arrow.

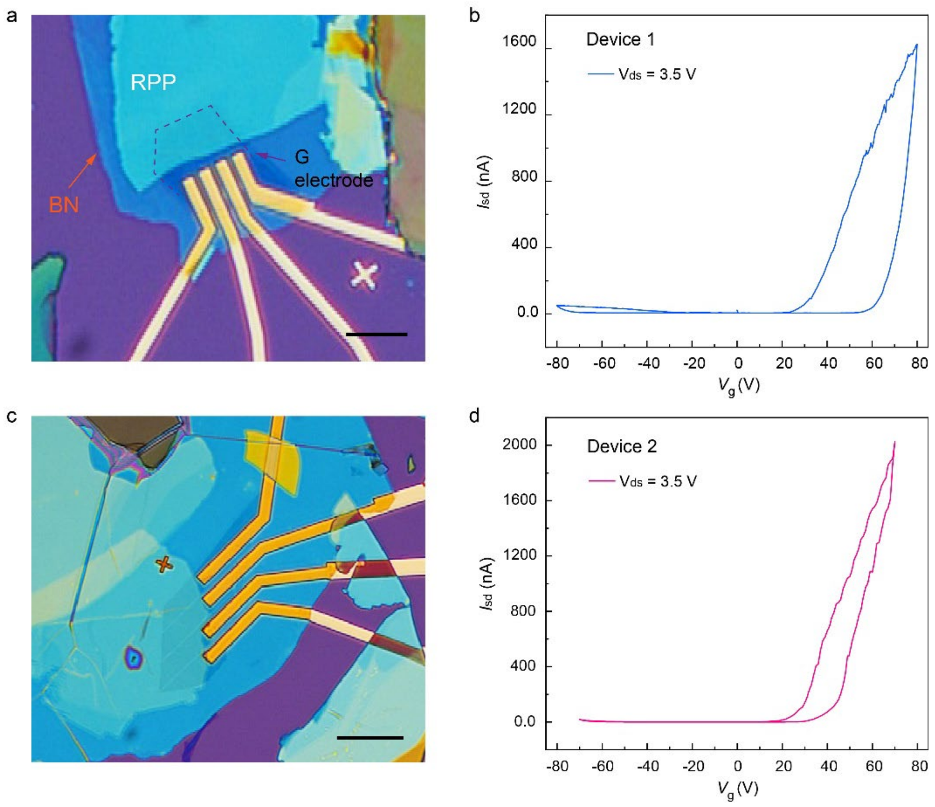

Supplementary Fig. 6 Two n=4 RPP FET devices by using graphene contact. a, Optical image of selected device one**.** Scale bar, 20 µm. **b**, Corresponding transfer characteristics of device one at 1.7 K. **c**, Optical image of selected device two. Scale bar, 20 µm. **d**, Corresponding transfer characteristic of device two at 1.7 K. The on-state current shows good repeatability, and that the field effect response is consistent with n-type device.

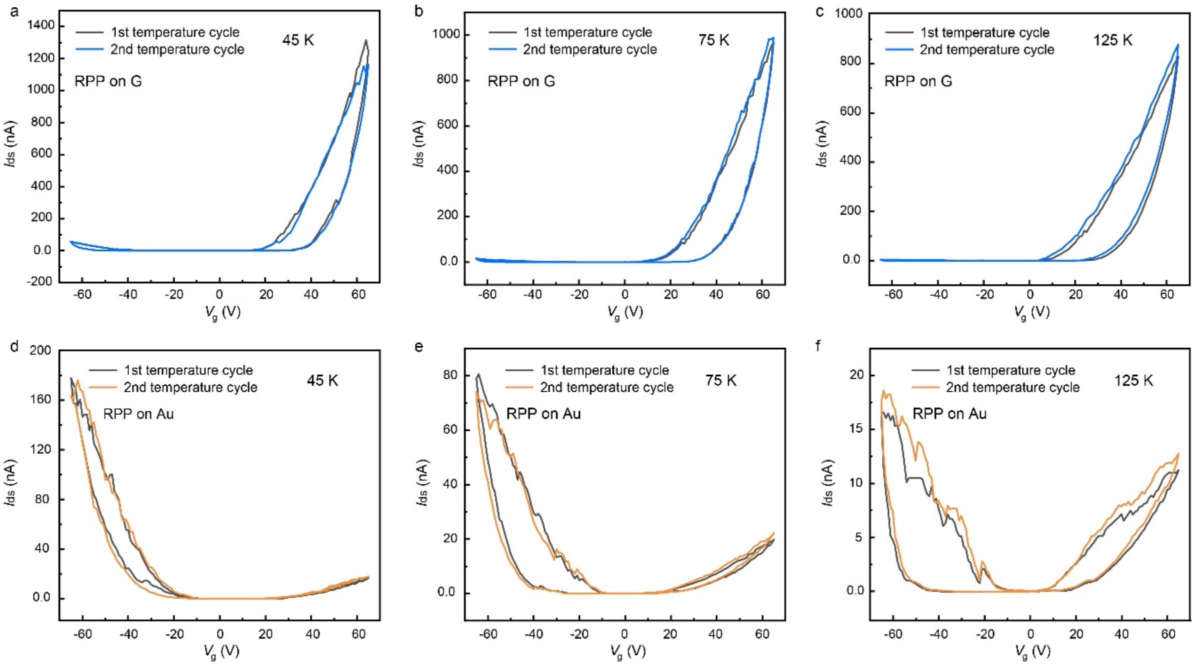

Supplementary Fig. 7 The operational stability of the 2D RPP (n = 4) FET device shown in Fig. 2 inset. a-c, Transfer characteristics upon thermal cycling measured at 45 K (**a**), 75 K (**b**) and 125 K (**c**) based on graphene contact. **d-f**, Transfer characteristics upon thermal cycling measured at 45 K (**d**), 75 K (**e**) and 125 K (**f**) based on Au contact.

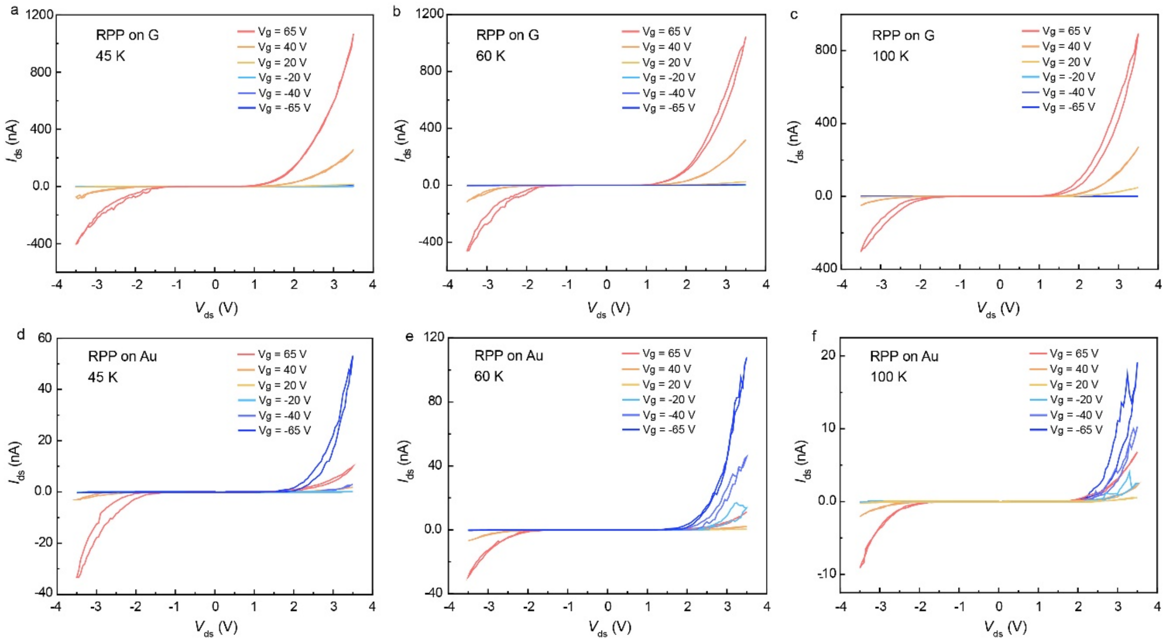

Supplementary Fig. 8 Comparative output curves of n=4 RPP/G and RPP/Au FETs at same temperature. *I*(*V*) output curves of RPP/G FET at **a**, 45 K; **b**, 60 K and **c**, 100 K. *I*(*V*) output curves of RPP/Au FET at **d**, 45 K; **e**, 60 K and **f**, 100 K.

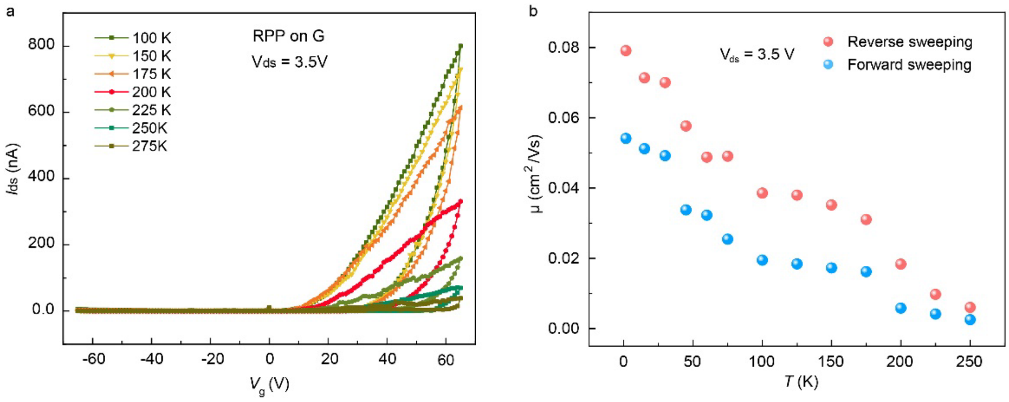

Supplementary Fig. 9 Temperature dependent transfer characteristics of n=4 RPP/G FETs. a, Temperature-dependent transfer characteristics from 100 to 275 K. **b**, Temperature dependent field-effect electron mobility extracted from **a** and **Fig. 3b**. In general, as temperature increases, the electron mobility decreases.

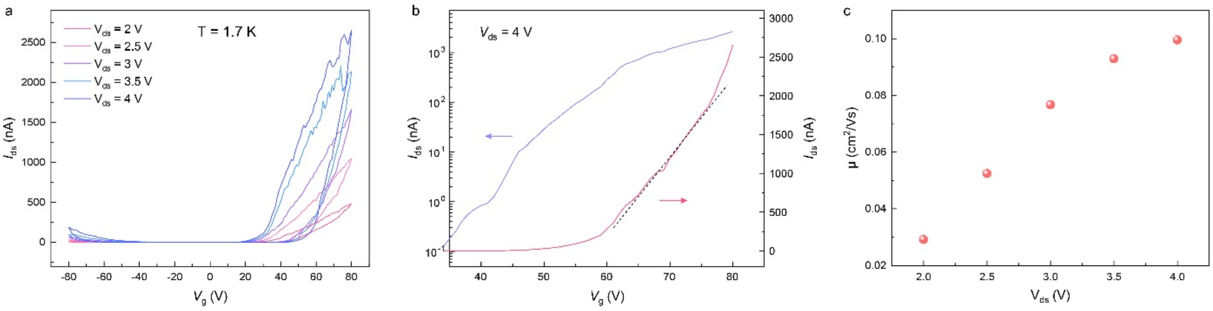

Supplementary Fig. 10 Mobility of RPP/G FETs. a, Transfer characteristics under different source-drain voltages. **b**, Representation of the transfer curve area for mobility calculation at V_{ds} = 4 V. **c**, Source-drain voltage dependent field effect electron mobility. The FET mobility increased as V_{ds} increased.

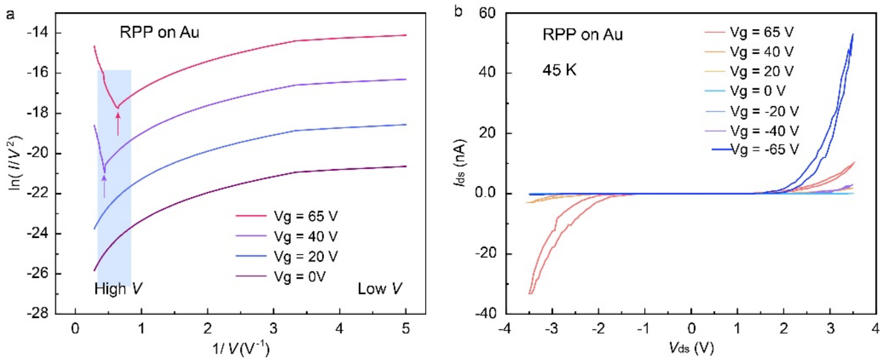

Supplementary Fig. 11 a. Fowler-Nordheim (F-N) plot of 2D RPP transistor with Au as electrode at 45 K, as a function of V_g. **b.** Output curves from which Fowler-Nordheim plot is extracted from in **a**.

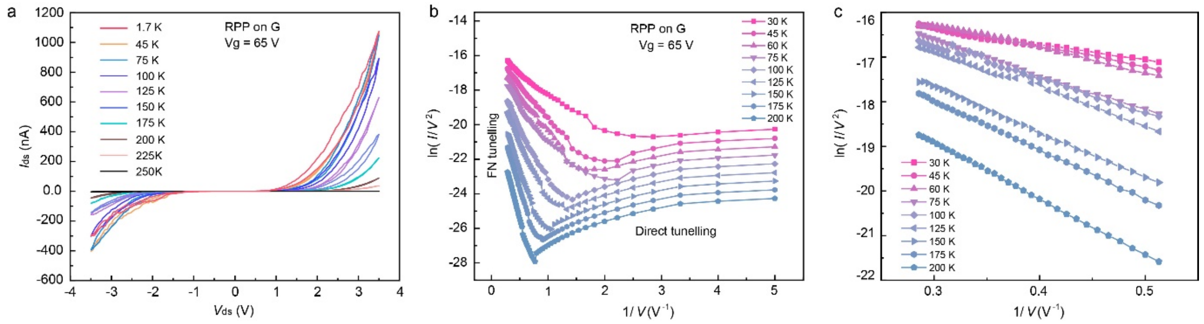

Supplementary Fig. 12 Temperature dependent charge transport characteristics of 2D RPP FETs with G contact. **a**, Output characteristics of FET at different temperature V_g = 65 V. **b**, Temperature dependent ln(I/V^2) versus 1/V plot. **c**, Magnified view of the ln(I/V^2) versus 1/V plot, showing the linear behaviour in agreement with Fowler-Nordheim tunnelling.

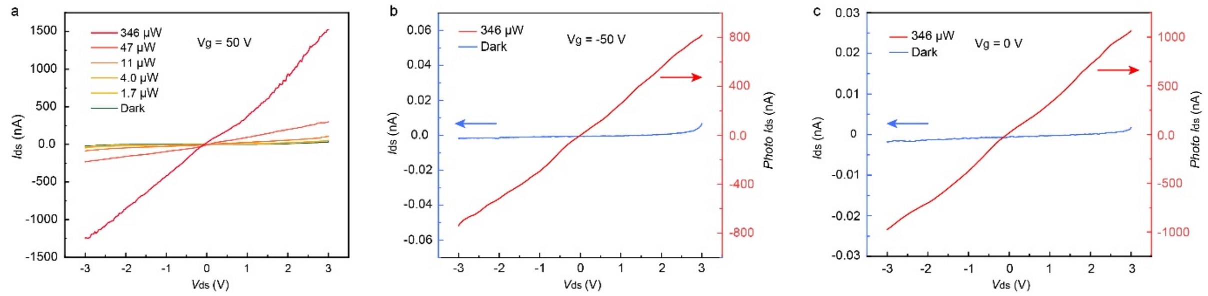

Supplementary Fig. 13 Characteristics of 2D RPP phototransistor for graphene contact. a, *I*-*V* curves of the phototransistor in the dark and under different illumination intensities at $V_g = 50$ V. **b** and **c**, Output curves of RPP phototransistor in the dark and under light illumination at $V_g = -50$ V (**b**) and $V_g = 0$ V (**c**).

Supplementary Table 1 A summary of reported FET works based on hybrid lead halide perovskites.

Material	Preparation method	L/W (μm)	S/D contact	Mobility (cm^2/Vs)	Regime for Mobility extraction	On/off ratio	Ref
**Bilayer (7 nm) $(C_4H_9NH_3)_2(CH_3NH_3)_3Pb_4I_{13}$**	Exfoliation	L=0.3 W=8.2	G	0.1 (e) @1.7 K	Linear	1.6×10^6	This work
**30 nm polycrystalline $(C_6H_5C_2H_4NH_3)_2SnI_4$**	Spin coating	L=28 W=1000	Pd, Pt, Au	0.6 (h) @RT	Saturation	10^4	[1]
**Polycrystalline $(PEA)_2SnI_4$**	Melt-process	L=105 W=1000	Au	1.7 (h) @RT	Saturation; Linear	10^6	[2]
**150 nm Polycrystalline $CH_3NH_3PbI_3$**	Spin coating	L=80/100 W=20000	Ni/Au	0.067/0.072 (e); 0.0066/0.021 (h) @78K	Linear/ saturation	10^5 @198 K	[3]
**100 nm ($CH_3NH_3PbI_3$); mixed-halide ($CH_3NH_3PbI_{3-x}Cl_x$)**	Spin coating	L=50 W=1000	Ti/Au	0.18(h)/0.17(e); 1.24(h)/1.0(e) @RT	Saturation	10^4	[4]
**$CH_3NH_3PbI_{3-x}Cl_x$**	Spin coating	L=30 W=1000	Au	1.3(h)/1(e) @RT	Saturation	10^2	[5]
**40 nm polycrystalline $PEASnI_4$**	Spin-coating	L=95/45 W=2000	Au	0.53 to 15 (h) @RT	Saturation	10^6	[6]
**Polycrystalline $CH_3NH_3PbI_3$; Single crystal $CH_3NH_3PbBr_3$**	Vapor grown; anti-vapor diffusion		Au	8 (μ_{hall}) 60 (μ_{hall}) @RT	Hall bar		[7]
**1 mm $CH_3NH_3PbBr_3$ single crystal**	Anti-vapor diffusion	L=750 W=1300	Ti	10 (μ_{hall}) @ 300 K	Hall bar		[8]
**40 nm Polycrystalline $(C_6H_5C_2H_4NH_3)_2SnI_4$**	Spin-coating	L=95 W=2000	Au, Ag, Al	0.6/1.1/1.5 (e) @RT	Saturation	5.2×10^4	[9]
**100 nm polycrystalline $CH_3NH_3PbI_3$**	Spin-coating	L=10 W=1000	Au	0.02 (e) @RT; 0.6 (e) @100 K; 0.05 (e) @270 K	Saturation	10^5	[10]
**250 nm $Cs_x(MA_{0.17}FA_{0.83})_{1-x}Pb(Br_{0.17}I_{0.83})_3$**	Spin-coating	L=20 W=1000	Au	2.1 (h) @RT 2.5 (e) @RT	Saturation	10^4	[11]
**MAPbX3 (X=Cl, Br, I)**	TSCs fabrication	L=265/240/ 185 W=50	Au	1.8 (h); 1.9 (h); 1.5 (h) @RT	Saturation	10^4; 10^3; 10^3	[12]
**400 nm polycrystalline $MAPbI_3$**	Hot-casting	L=70 W=100	Au	0.001 (h) @RT	Saturation	10^4	[13]
**$RbCsFAMAPbI_3$**	Spin coating	L = 100 W = 1000	Cr/Au	1.2 (e) @RT	Saturation	10^4	[14]
**$(PEA)_2SnI_4$**	Spin coating	L=200 W=1000	Au	3.51 (h)	Saturation	3.4×10^6	[15]
**$(4Tm)_2SnI_4$**	Spin coating	L=40 W=2880	Au	2.32 (h,max)	Linear	10^5-10^6	[16]

Supplementary Table 2 The slope result by linear fitting for the Fowler-Nordheim tunnelling regime at different V_g.

V_g (V)	Slope
20	-6.39 ± 0.26
40	-5.36 ± 0.03
65	-3.98 ± 0.03

Supplementary References

1 Kagan, C. R., Mitzi, D. B. & Dimitrakopoulos, C. D. Organic-Inorganic Hybrid Materials as Semiconducting Channels in Thin-Film Field-Effect Transistors. *Science* **286**, 945 (1999).

2 Mitzi, D. B. *et al.* Hybrid Field-Effect Transistor Based on a Low-Temperature Melt-Processed Channel Layer. *Adv. Mater.* **14**, 1772-1776 (2002).

3 Chin, X. Y., Cortecchia, D., Yin, J., Bruno, A. & Soci, C. Lead iodide perovskite light-emitting field-effect transistor. *Nat. Commun.* **6**, 7383 (2015).

4 Li, F. *et al.* Ambipolar solution-processed hybrid perovskite phototransistors. *Nat. Commun.* **6**, 8238 (2015).

5 Mei, Y., Zhang, C., Vardeny, Z. V. & Jurchescu, O. D. Electrostatic gating of hybrid halide perovskite field-effect transistors: balanced ambipolar transport at room-temperature. *MRS Commun.* **5**, 297-301 (2015).

6 Matsushima, T. *et al.* Solution-Processed Organic–Inorganic Perovskite Field-Effect Transistors with High Hole Mobilities. *Adv. Mater.* **28**, 10275-10281 (2016).

7 Chen, Y. *et al.* Extended carrier lifetimes and diffusion in hybrid perovskites revealed by Hall effect and photoconductivity measurements. *Nat. Commun.* **7**, 12253 (2016).

8 Yi, H. T., Wu, X., Zhu, X. & Podzorov, V. Intrinsic Charge Transport across Phase Transitions in Hybrid Organo-Inorganic Perovskites. *Adv. Mater.* **28**, 6509-6514 (2016).

9 Matsushima, T. *et al.* N-channel field-effect transistors with an organic-inorganic layered perovskite semiconductor. *Appl. Phys. Lett.* **109**, 253301 (2016).

10 Senanayak, S. P. *et al.* Understanding charge transport in lead iodide perovskite thin-film field-effect transistors. *Sci. Adv.* **3**, e1601935 (2017).

11 Yusoff, A. R. b. M. *et al.* Ambipolar Triple Cation Perovskite Field Effect Transistors and Inverters. *Adv. Mater.* **29**, 1602940 (2017).

12 Yu, W. *et al.* Single crystal hybrid perovskite field-effect transistors. *Nat. Commun.* **9**, 5354 (2018).

13 Canicoba, N. D. *et al.* Halide Perovskite High-k Field Effect Transistors with Dynamically Reconfigurable Ambipolarity. *ACS Mater. Lett.* **1**, 633-640 (2019).

14 Senanayak, S. P. *et al.* A general approach for hysteresis-free, operationally stable metal halide perovskite field-effect transistors. *Sci. Adv.* **6**, eaaz4948 (2020).

15 Zhu, H. *et al.* High-Performance and Reliable Lead-Free Layered-Perovskite Transistors. *Adv. Mater.* **32**, 2002717 (2020).

16 Gao, Y. *et al.* Highly Stable Lead-Free Perovskite Field-Effect Transistors Incorporating Linear π-Conjugated Organic Ligands. *Journal of the American Chemical Society* **141**, 15577-15585 (2019).
